# SXP01: a novel bacteriophage for combating *Shewanella xiamenensis* in aquaculture

**DOI:** 10.3389/fmicb.2025.1652450

**Published:** 2025-10-16

**Authors:** Chen Li, Yangjun Zhang, Yuyuan Peng, Guofan Zhang, Jiajie Xie, Xinran Long, Linger Qian, Yibo Hu, Shengbiao Hu

**Affiliations:** Hunan Provincial Key Laboratory of Microbial Molecular Biology, College of Life Science, Hunan Normal University, Changsha, China

**Keywords:** *Shewanella xiamenensis*, bacteriophage, holin-independent lysis system, phage therapy, aquaculture

## Abstract

**Introduction:**

The emergence of multi-drug resistant *Shewanella xiamenensis* poses a significant threat to aquaculture and public health, driving the need for alternative therapies like bacteriophages.

**Methods:**

A novel bacteriophage, SXP01, was isolated using S. xiamenensis A001 as a host. Its kinetic parameters (MOI, latent period) and stability across temperature (4-60°C), pH (6-11), and chloroform were characterized. The lysis cassette was identified genomically, and a recombinant endolysin (LysSXP-1) was expressed to test lytic activity. In vivo efficacy was evaluated in a crucian carp infection model.

**Results:**

SXP01, a Myoviridae member, showed an optimal MOI of 0.1, a 20-minute latent period, and high environmental stability. Its lysis cassette encodes two endolysins, with LysSXP-1 demonstrating direct lytic activity. In the fish model, SXP01 treatment significantly increased survival rates from 13.3% to 56.7%.

**Discussion:**

The study identifies SXP01 as a highly stable and effective bacteriophage. The synergistic action of its endolysins and its demonstrated in vivo efficacy highlight its strong potential as a targeted biocontrol agent to manage *S. xiamenensis* infections in aquaculture sustainably.

## 1 Introduction

*Shewanella* is a facultative anaerobic bacterium belonging to the family *Shewanellaceae* within the order Gammaproteobacteria, primarily isolated from diverse environments, including freshwater and marine ecosystems (Mukherjee et al., 2020; Hau and Gralnick, 2007; São-José et al., 2000). *Shewanella xiamenensis* (*S. xiamenensis*), an emerging zoonotic pathogen, was first reported in coastal sediments in Xiamen, China (Huang et al., 2010). This bacterium is prevalent in aquatic ecosystems worldwide and frequently acts as a pathogen in aquaculture (Jiang et al., 2023). It has also been identified as the causative agent of intra-abdominal infections and intestinal colonization in humans (Liu et al., 2022; Potron et al., 2011). *S. xiamenensis* is posing a threat to aquaculture, capable of causing sepsis in fish, including varying degrees of damage to tissues and organs, even leading to fatality (Chen et al., 2025). Furthermore, this bacterium has shown resistance to multiple antibiotics, which undermines the common practice of using commercial antibiotics to manage bacterial infections in aquaculture. This resistance also raises public health concerns, as it could potentially be transmitted to humans through the food chain (Desbois et al., 2020; Wang et al., 2024; Huang et al., 2019; Johnson et al., 2025; Zhu et al., 2022). Consequently, there is an urgent need to further investigate and develop novel strategies to combat *S. xiamenensis* infections.

Bacteriophages, recognized as the most diverse life forms on earth, exhibit a high degree of host specificity and can rapidly lyse bacteria, including drug-resistant strains, without impacting other bacterial populations. This characteristic underscores their significant potential for controlling pathogens in aquaculture (Ramos-Vivas et al., 2021). To date, only 21 infectious *Shewanella* phages with complete nucleotide genomes have been documented in the NCBI virus database. This number is considerably lower compared to the studies of phages that target other bacteria in aquaculture, such as those infecting *Aeromonas hydrophila* and *Aeromonas salmonicida* (Chen et al., 2024). However, studies have shown that in aquaculture, bacteriophages can consistently and effectively control the growth of *Shewanella*, including *Shewanella putrefaciens* and *Shewanella algae*, demonstrating strong biocontrol capabilities (Liu et al., 2025; Li et al., 2025). Therefore, screening for novel bacteriophages targeting *S. xiamenensis*, an indispensable member of the *Shewanella* genus, is of significant importance.

Generally, there are two widely accepted lysis mechanisms in bacteriophages: the holin-endolysin system and the signal arrest release (SAR)-endolysin system. In the canonical holin-endolysin system, the holin protein accumulates in the inner membrane during phage assembly, forming a hydrophilic cross-channel that allows endolysin to traverse the inner membrane and lyse the cell wall (White et al., 2011). Conversely, the SAR endolysin contains SAR sequences for signal-arrest-release, enabling it to embed itself in the inner membrane and remain inactive until its accessory proteins, known as pinholins, induce membrane depolarization, thereby releasing endolysin into the periplasm (Sun et al., 2009). Both mechanisms require spanins to facilitate the penetration of endolysin through the outer membrane in Gram-negative bacteria (Young, 2014). However, studies have demonstrated that endolysin can be secreted through pathways independent of holin action, such as via its own signal peptide (São-José et al., 2000), transmembrane domain (Bai et al., 2020), or with the assistance of chaperone proteins (Catalão et al., 2010). Endolysin, also referred as lysin, is a peptidoglycan hydrolase encoded by double-stranded DNA phages that functions by degrading the peptidoglycan layer of the bacterial cell wall, thereby facilitating the release of viral particles (Cahill and Young, 2019). Endolysin can be classified into five categories based on the cleavage site: N-acetyl cytosolic amidase, transglycosylase, N-acetyl aminoglucosidase, endopeptidase, and N-acetyl cytosolic acyl-L-alanine amidase (Catalão et al., 2013). Holin is a class of small-molecule membrane proteins characterized by at least one predicted transmembrane structural domain, which significantly influences the duration of the phage infection cycle (Ahammad et al., 2020). Furthermore, holin accumulates in the cell membrane, triggering its disruption (Bednarek et al., 2022). This disruption causes a loss of the proton motive force and depolarization of the cell membrane, ultimately resulting in the release and activation of endolysin, which leads to the rapid lysis of the host bacteria (Fernandes and São-José, 2016).

In this study, we isolated and characterized a virulent phage (designated as SXP01) infecting *S. xiamenensis* from the sewage of Meixi Lake, Changsha, Hunan province, China. The biological properties and complete genome sequence of the SXP01 phage were analyzed. The lysis cassette of SXP01, which comprises LysSXP-1 and LysSXP-2, was identified within the genome. Furthermore, we explored the protective effect of SXP01 in crucian carp infected with *S. xiamenensis*, the results of which indicate its great potential for controlling fish diseases.

## 2 Materials and methods

### 2.1 Samples, bacterial strains and growth conditions

Sewage samples were sourced from Meixi Lake in Changsha, Hunan province, China. *S. xiamenensis* A001, previously isolated from diseased fish, was cultivated on Luria-Bertani (LB) at 30 °C. *E. coli* DH5α and BL21 (DE3) were grown on LB medium at 37 °C, with appropriate antibiotics (100 μg/mL ampicillin, 50 μg/mL kanamycin) added as required.

### 2.2 Isolation and purification of phage

A single colony of *S. xiamenensis* A001 was inoculated into LB medium and shaken overnight at 30 °C. Sewage from Meixi Lake in Changsha was collected, and 40 mL of filtered water samples were added to 20 mL of triple beef paste peptone medium. Subsequently, 200 μL of the host bacterium (1 × 10^9^ CFU/mL) was introduced and mixed in a shaking incubator at 30 °C for overnight incubation. After centrifugation, the supernatant was collected, and 200 μL of the supernatant solution was mixed with the indicator bacteria and incubated in a shaker at 30 °C for 12 h. Following centrifugation, the supernatant was filtered through a 0.22 μm filter. A 10 μL drop of the filtrate was placed on a plate covered with the indicator bacteria, and the plaque was observed. Phages were purified using the double agar plate method, and purification was repeated four to five times until a single plaque morphology was observed (White et al., 2011; Carroll-Portillo et al., 2021). The host range was tested with the same method. Information regarding the tested strains is shown in Table 1.

**Table 1:** The detection of host range of phage SXP01.

Bacterial strain	Spot of SXP01^a^	Strain resource
*Shewanella xiamenensis A001*	+	lab collections
*Edwardsiella tarda*	−	lab collections
*Citrobacter freundii*	−	lab collections
*Aeromonas harophila*	−	lab collections
*Aeromonas salmonicida*	−	lab collections
*Aeromonas veronii*	−	lab collections
*Erwinia tasmaniensis*	−	lab collections
*Plesiomonas shigelloides*	−	lab collections

a + indicates that plaques were observed; − indicates that plaques were not observed.

### 2.3 Transmission electron microscopy

A 15 μL aliquot of phage suspension was applied to a copper grid with a supporting film and negatively stained with 2% phosphotungstic acid. The specimens were examined using a transmission electron microscope (Hitachi HT7700, Tokyo, Japan) at an acceleration voltage of 80 kV.

### 2.4 Determination of multiplicity of infection

The multiplicity of infection (MOI) is defined as the ratio of phages to host bacteria at the onset of infection. Equal volumes of SXP01 solution and *S. xiamenensis* A001 were mixed in sterile LB medium at varying MOI levels ranging from 0.01 to 100 and incubated for 4 h at 30 °C. Following incubation, the mixture was then centrifuged to remove free phages, and the phage titer was subsequently determined. The infection complex exhibiting the highest titer was selected as optimal. All experiments were conducted in triplicate to ensure reliability.

### 2.5 One-step growth curve

One-step growth curve assays were performed as described previously (Wang et al., 2022). Briefly, fresh *S. xiamenensis* A001 culture (10^9^CFU/mL) was mixed with phage at the MOI of 0.1 for 15 min at 30 °C. Then the culture was centrifuged at 9000 rpm for 10 min to remove unadsorbed phages and resuspended in 100 mL LB, and the dilution were incubated at 30 °C. Samples were taken every 10 min and plated using the double-layer agar plate method to determine phage titer. Each assay was performed in triplicate.

### 2.6 Phage physiological characteristics quantification

Phage filtrate (1.7 × 10^9^ PFU/mL) was subjected to different temperatures ranging from 4 to 60 °C for 1 h. Subsequently, the phage was then mixed with logarithmic phase host bacteria at an MOI of 0.1. The phage titer at different temperatures was determined using the double-layer agar plate method, with each temperature tested in triplicate. Additionally, phage filtrate (1.7 × 10^9^ PFU/mL) was mixed with LB solution at specific pH levels ranging from 1 to 14 and incubated for 1 h. The phage was then combined with host bacteria in exponential phase at an MOI of 10, and the phage titer at various pH values was assessed using the double-layer agar plate method, with each pH tested in triplicate. Phage filtrate (1.7 × 10^9^ PFU/mL) was incubated with LB containing varying concentrations of chloroform (1, 2, and 5%) for 1 h. The phage was mixed with logarithmic phase host bacteria at an MOI of 10, and the titers were determined using the double-layer agar plate assay for different salt concentrations, with three repetitions for each concentration.

### 2.7 Sequencing, annotation and bioinformatics analysis of functional proteins of SXP01

DNA from phage SXP01 was extracted using a *λ* phage genome extraction kit and subsequently sequenced at Panoson Biologicals. The Rast Online database was employed for genomic annotation, while CG view was utilized to generate a genome map (Grant et al., 2023). Phylogenetic analysis was performed using MEGA11 with the neighbor-joining method and Clustal alignment (Tamura et al., 2021). The BLASTp program facilitated the comparison of protein sequences encoded by the phage SXP01 gene against the NR database. The positions of LysSXP-1 and LysSXP-2 were analyzed with Snapgene, and their signal peptides were predicted by SignaIP 5.0 Server. Transmembrane domains (TMDs) were predicted utilizing TMHMM Server v.2.0. The genome sequence data of phage SXP01 have been submitted to the GenBank databases under accession number PV593634.

### 2.8 Evaluation of lytic activity of lysis cassette

The ORFs of lysis cassette (LysSXP-1 and LysSXP-2) were cloned into pET28a vector and transformed into *E. coli* DH5α separately (Primer sequences are shown in Supplementary Table S1). The recombinant expression vectors were sequenced and then transformed into *E. coli* BL21 (DE3). The overnight culture of positive transformant was inoculated into 20 mL fresh LB medium at a ratio of 1% and shaken at 37 °C for 2.5 h (until OD600 = 0.5–0.7). IPTG was added to a final concentration of 0.5 mmol/L to induce protein expression for 4 h at 37 °C. During this period, bacterial fluid samples were collected hourly, and OD600 values were measured to evaluate the growth effects on *E. coli*. After 1 h of induced expression, samples were washed three times with phosphate buffer solution (PBS) and resuspended. A mixture of 1 mL of bacterial solution, 1.5 μL of SytoTM9 Green Fluorescent Nucleic Acid Staining Reagent, and 1.5 μL of propidium iodide was incubated for 30 min, after which 10 μL of the *E. coli* staining solution was examined under a fluorescence microscope.

### 2.9 Protective effect of phage SXP01 on the crucian carp *Shewanella xiamenensis*

A total of 120 healthy and uniformly sized crucian carp were randomly assigned to four groups, each with three replicates. The water used throughout the experiment was aerated to eliminate chlorine. In the PBS group, 100 μL of PBS was intraperitoneally injected into the crucian carp, followed by an additional 100 μL of PBS 3 h later. In experimental group 1, 100 μL of phage (1.7 × 10^8^ PFU/mL) was intraperitoneally injected into the crucian carp, followed by another 100 μL of phage 3 h later. In Experimental group 2, 100 μL of host bacteria (1 × 10^9^ CFU/mL) was intraperitoneally injected into the crucian carp, followed by 100 μL of PBS 3 h later. In Experimental group 3, 100 μL of host bacteria (1 × 10^9^ CFU/mL) was intraperitoneally injected into the crucian carp, followed by 100 μL of phage (1.7 × 10^8^ PFU/mL) 3 h later. The experiment lasted for 14 days, during which the survival rates of the crucian carp were recorded for each group. Three days post-injection, one crucian carp from each group was dissected to collect kidney, liver, and intestinal tissues. These tissues were fixed in 4% paraformaldehyde and sent to Wuhan Sevier Biotechnology Co. Ltd. for histopathological analysis, which included embedding, slicing, and H&E staining.

### 2.10 Ethics statement

The animal study was approved by Hunan Normal University Biomedical Research Ethics Committee (2024084). The study was conducted in accordance with the local legislation and institutional requirements.

### 2.11 Statistical analysis

Statistical analysis of bar chart was performed using GraphPad Prism v8.0.2 (GraphPad Software, USA).

## 3 Results

### 3.1 Isolation and characterization of bacteriophage SXP01

**Figure 1:**
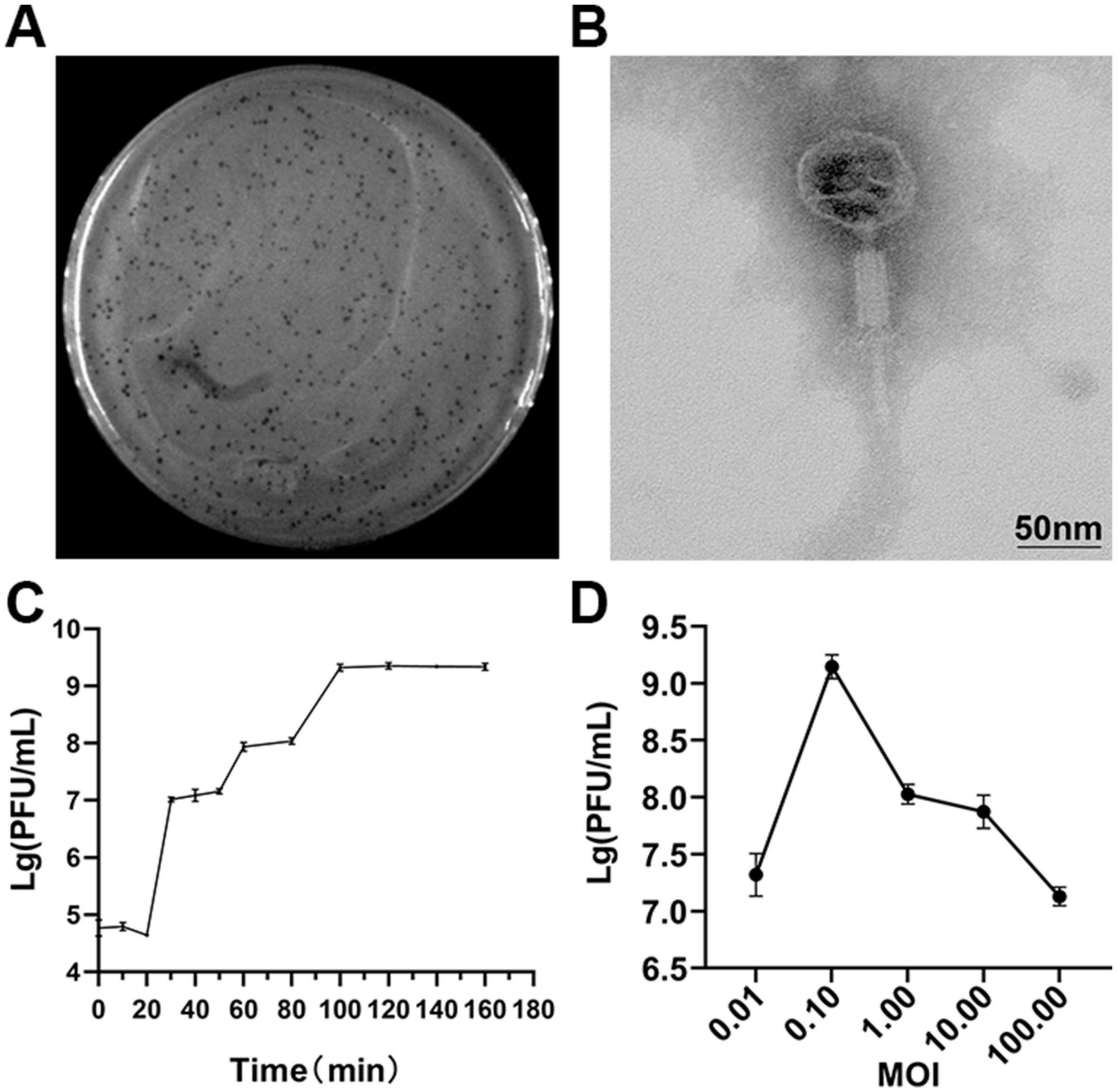
Isolation, morphology and biological characteristics of SXP01. **(A)** Plaques formation of SXP01 on a double agar plate that exhibited a regular round shape, uniform size (~2 mm in diameter), translucency, and the absence of a halo ring at the periphery after four to five purification cycles. **(B)** Transmission electron micrograph showing phage morphology, which reveals an icosahedral head with a diameter of approximately 55 ± 2 nm and a flexible tail measuring approximately 117 ± 5 nm. Bar graph, 50 nm. **(C)** One-step growth curve determination of SXP01 phage. **(D)** Determination of optimal multiplicity of infection (MOI) of phage of SXP01.

A potent phage strain, designated SXP01, was isolated using the fish pathogen *S. xiamenensis* A001 as its host. The phage was purified utilizing the double-layer agar plate method, resulting in phage plaques that exhibited a regular round shape, uniform size (~2 mm in diameter), translucency, and the absence of a halo ring at the periphery after four to five purification cycles (Figure 1A). To determine the host range of SXP01, we tested some common fish pathogens isolated in our laboratory from aquaculture, including some Aeromonas species, *Plesiomonas shigelloides*, and *Edwardsiella tarda*. The results indicated that SXP01 possesses a narrow host spectrum, specifically infecting only its host bacterium, *S. xiamenensis* A001, while demonstrating no lytic effect on other tested pathogens (Table 1). This specificity allows SXP01 to minimize damage to other probiotics in the environment when employed as an antibacterial agent. Electron microscopy revealed that the phage features an icosahedral head with a diameter of approximately 55 ± 2 nm and a flexible tail measuring approximately 117 ± 5 nm (Figure 1B). Based on its morphological characteristics, phage SXP01 was classified within the family Myoviridae. A one-step growth curve experiment was conducted to elucidate the various stages of the SXP01 phage life cycle during infection. The results indicated that the latent period for SXP01 was 20 min, while the burst period lasted for 100 min, and the average burst size of this phage was about 215 phages/cell. (Figure 1C). At a multiplicity of infection (MOI) of 0.1, the phage titer reached its peak value of approximately 10^9^ PFU/mL (Figure 1D), significantly higher than in other groups, suggesting that the optimal MOI for bacteriophage SXP01 is 0.1.

### 3.2 Thermal, pH, and chloroform stability of SXP01 lytic activity

**Figure 2:**
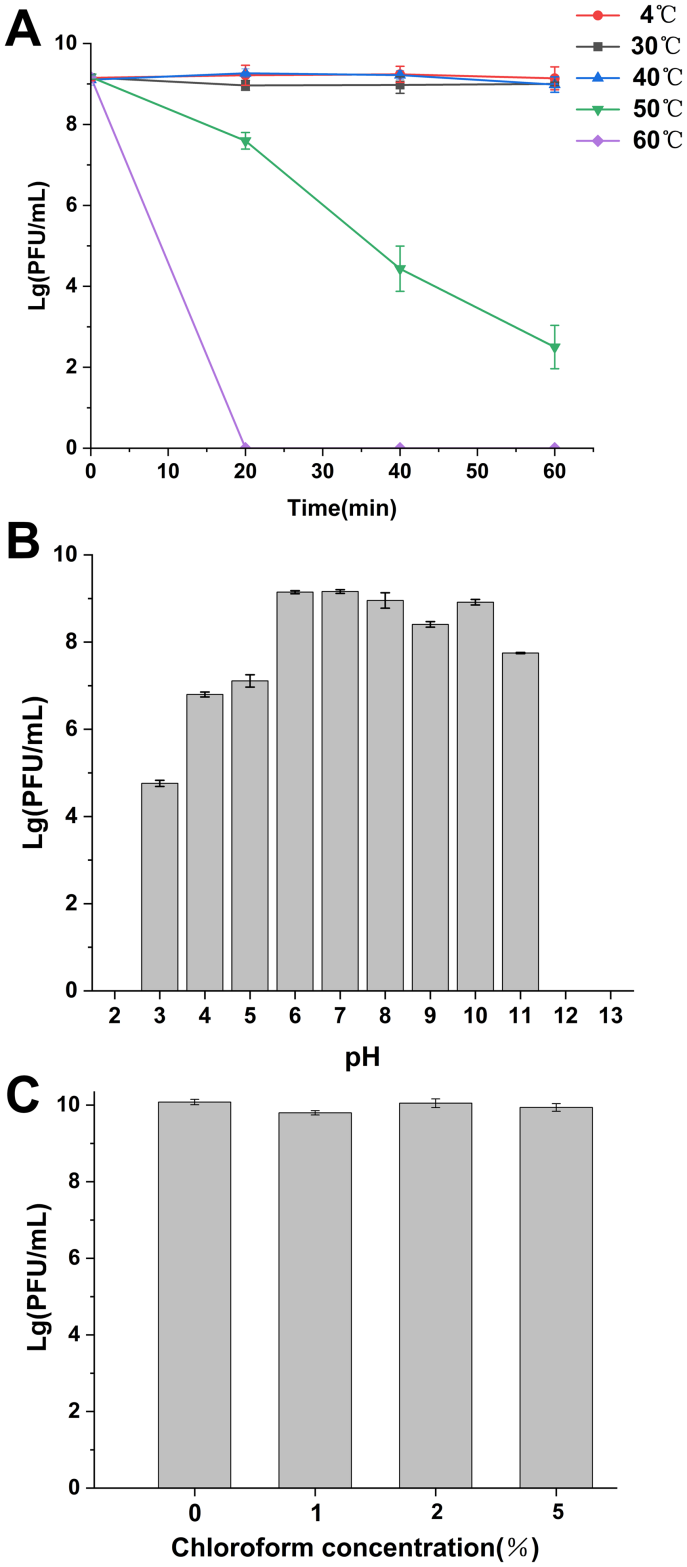
Survival and stability of SXP01. **(A)** SXP01 temperature stability analysis (4 ~ 60 °C). **(B)** SXP01 pH stability analysis (1–14). **(C)** Chloroform sensitivity analysis (1–5%).

The lytic activity of SXP01 was observed to remain stable and consistent after undergoing incubation for a duration of 1 h at varying temperature conditions ranging from 4 °C to 60 °C, the titer of phage can reach to 10^9^ PFU/mL. The bacteriophage viability gradually decreased at 50 °C, and the phage titer dropped to 10^2^ PFU/mL after 1 h. Whereas, at 60 °C, the bacteriophages could not survive (Figure 2A). SXP01 is significantly affected under acidic conditions, its cleavage activity continuously decreases at pH ≤ 5, reaching the lowest titer of 10^2^ PFU/mL at pH 3. Conversely, the phage remains relatively stable within the pH range of 6–11, with the titer consistently maintained around 10^8^ PFU/mL (Figure 2B). Interestingly, SXP01 demonstrated good activity and resilience when exposed to a range of chloroform concentrations, as shown in Figure 2C, indicating that the phage SXP01 lacks an envelope structure, and its capsid and tail fibers do not contain lipids or lipoproteins.

### 3.3 Genomic characterization and phylogenetic analysis of SXP01

**Figure 3:**
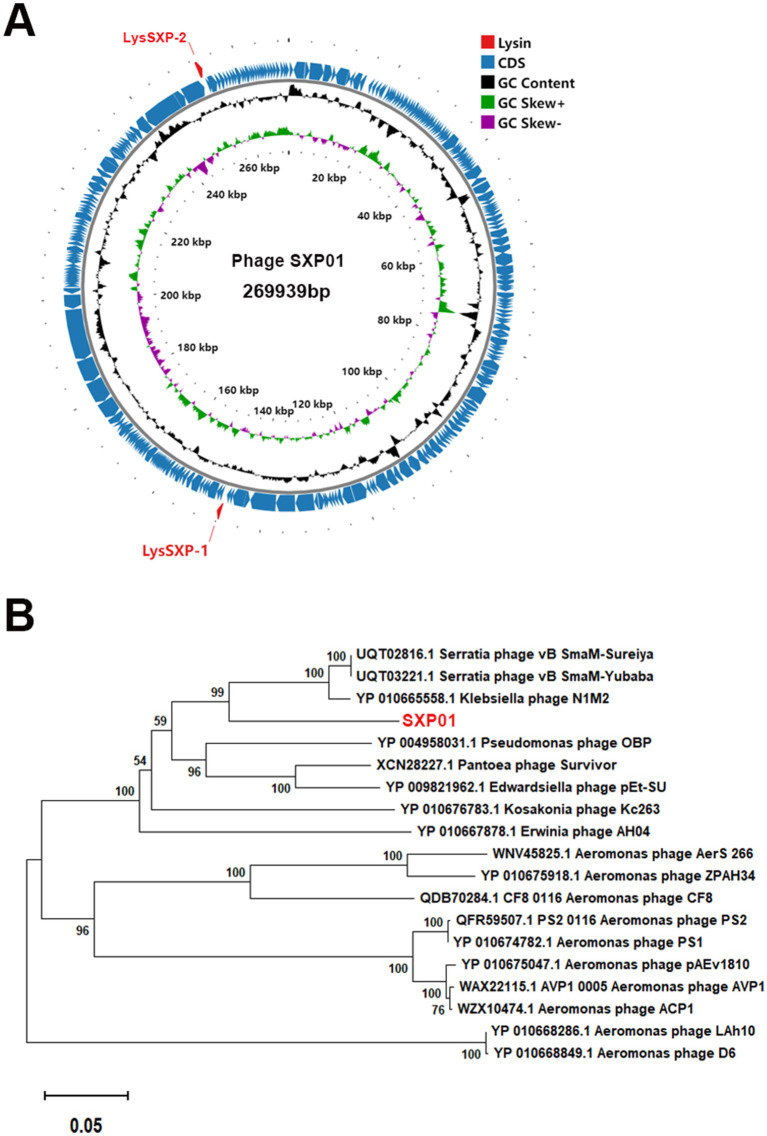
Genome sequencing analysis of the phage SXP01. **(A)** Graphical representation of the phage SXP01. Circles show (from the outside to the inside) (1) localisation of lysis cassette, red font; (2) coding sequences and orientation; (3) G + C% content, values greater than 42.99% are on the outside, values less than 42.99% are on the inside; (4) GC skew, values greater than zero are in green, while those lower than zero are in purple; (5) physical map scaled in kb. **(B)** Neighbor−joining phylogenetic tree based on the major capsid protein sequence of phage SXP01.

The entire genome of phage SXP01 is comprised of a circular double-stranded DNA, with a total length of 269,939 bp and a GC content of 42.99% (Figure 3A). The genome of phage SXP01 contains 283 open reading frames (ORFs), which account for approximately 94.25% of its total length. Among these 283 ORFs, 81 were successfully annotated regarding their functions; specifically, ORF151 encodes a putative glycosyltransferase (LysSXP-1), and ORF257 encodes a putative endolysin (LysSXP-2). To elucidate the evolutionary status of phage SXP01, the amino acid sequence of its major capsid protein was used for phylogenetic analysis, as this protein is generally conserved among similar phages (Stamereilers et al., 2018). The genome of SXP01 exhibited 83.68% identity with the Klebsiella phage N1M2, which aligns with the phylogenetic cluster derived from the entire genome (Figure 3B), indicating that SXP01 is a novel phage.

### 3.4 Structural and functional divergence of LysSXP01 and LysSXP-2 in SXP01

**Figure 4:**
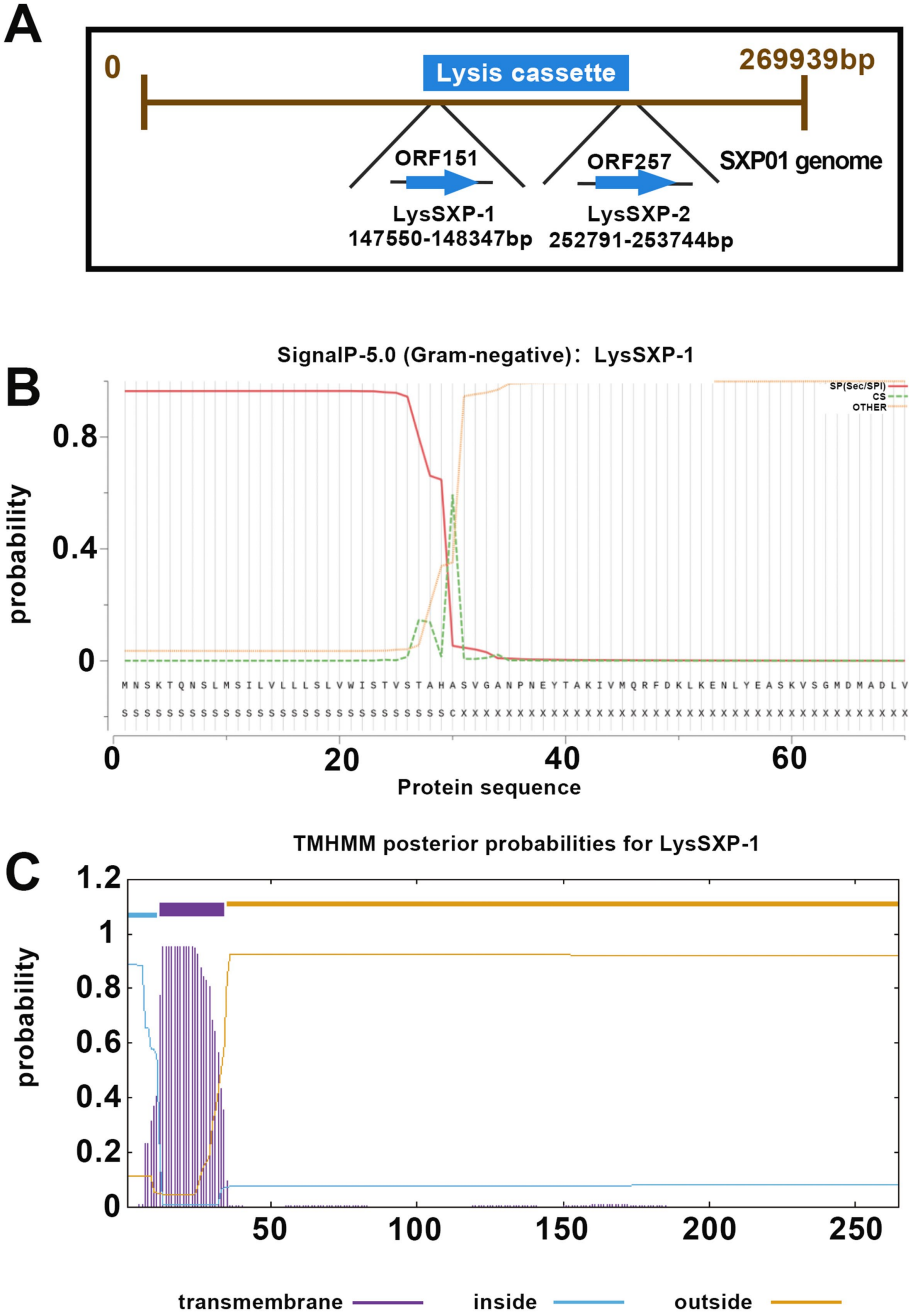
Bioinformatic analysis of lysin proteins of the SXP01. **(A)** Locations of LysSXP-1 and LysSXP-2 in the genome of phage SXP01. **(B)** Signal peptide prediction of LysSXP-1. **(C)** Transmembrane domain prediction of LysSXP-1.

The SXP01 genome was predicted to encode two lysin proteins, ORF151 encoded a putative glycosyltransferase (LysSXP01), which was located at 147550–148347 bp in the genome, and ORF257, located at 252791–253744 bp, encoded a putative endolysin (LysSXP02) (Figure 4A). Signal peptides (SP) and TMHMM analysis indicated that LysSXP01 had a transmembrane domain and a SP was annotated at the N-terminal 30 amino acids while LysSXP-2 did not contain a signal peptide with a transmembrane domain (TMD) (Figures 4B,C). Further analysis of these proteins revealed that LysSXP01, with its transmembrane domain, is likely to be anchored to the cell membrane, playing a crucial role in the cell wall degradation process. On the other hand, LysSXP-2, lacking a signal peptide and TMD, is expected to function as a soluble enzyme within the cell, contributing to the breakdown of the peptidoglycan layer. The distinct locations and functions of these two proteins suggest a coordinated mechanism for lysin activity within the SXP01 genome, potentially playing a significant role in the bacteriophage’s lifecycle and host cell lysis. The presence of a membrane-anchored lysin, LysSXP01, suggests that it could be involved in initial breaches of the cell wall, creating entry points for the soluble lysin, LysSXP-2, which would then act on the peptidoglycan layer to complete the degradation process. This dual strategy ensures a more effective and rapid lysis of the host cell, which is critical for the propagation of the bacteriophage. The understanding of these mechanisms not only sheds light on the bacteriophage-host interaction but also opens up potential avenues for the development of novel antibacterial strategies that could target these specific lysin proteins.

### 3.5 Heterologous expression and functional characterization of LysSXP-1 and LysSXP-2 in *Escherichia coli*

**Figure 5:**
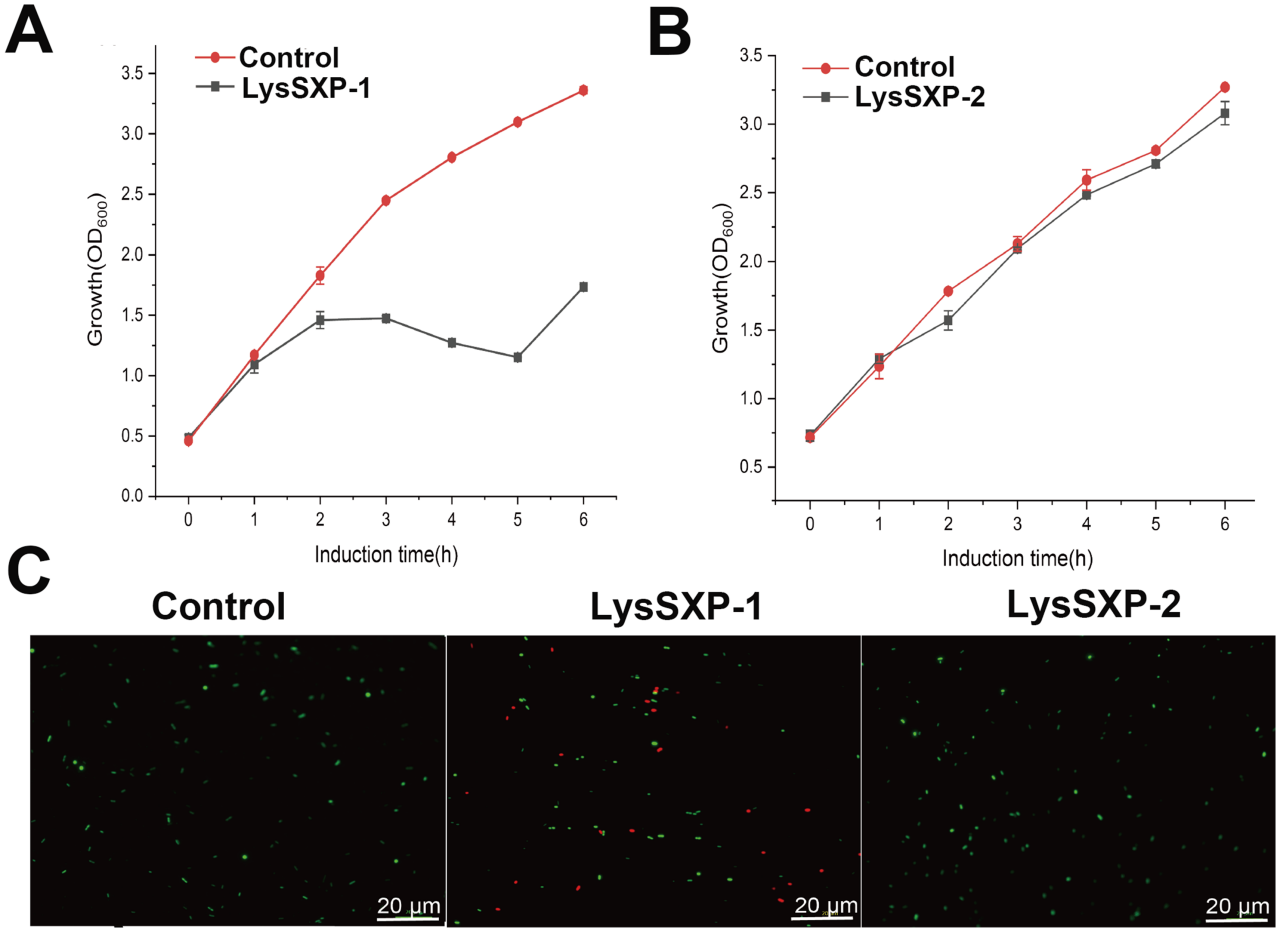
Functional analysis of LysSXP-1 and LysSXP-2. Effect of LysSXP-1 and LysSXP-2 protein expression on the growth **(A,B)** and cell viability **(C)** of *E. coli*. Green and red fluorescence represent live and dead cells, respectively Scale bar = 20 μm.

The ORFs encoding LysSXP-1 and LysSXP-2 were separately cloned into the pET28a vector and subsequently heterologously expressed in *E. coli* BL21 (DE3) to investigate their lytic activity. The expression of the target proteins was meticulously analyzed using sodium dodecyl sulfate polyacrylamide gel electrophoresis (SDS-PAGE). LysSXP-1 was observed to be approximately 30 kDa (Supplementary Figure S1A), while LysSXP-2 was approximately 35 kDa (Supplementary Figure S1B), both of which were in agreement with the expected molecular weights. During the expression of LysSXP-1, a phenomenon of cell lysis was observed, which resulted in a significant reduction in the bacterial count, as evidenced by a notable decrease in optical density at 600 nm (Figure 5A). Interestingly, LysSXP-1 was only detected in the precipitate of the cell lysate (as shown in Supplementary Figure S1A). In contrast, the cell density of *E. coli* was observed to increase over time during the expression of LysSXP-2 (Figure 5B), suggesting that LysSXP-2 does not exhibit lytic activity. Following 1 h of induction, the cells were subjected to staining with SytoTM9 green fluorescent nucleic acid stain and propidium iodide stain (where green fluorescence signifies live bacteria, and red fluorescence signifies dead bacteria). The outcomes of this staining procedure revealed that certain strains harboring the plasmid pET28a-LysSXP-1 exhibited red fluorescence, indicating that LysSXP-1 is indeed toxic to *E. coli*, whereas all strains carrying the plasmid pET28a-LysSXP-2 displayed green fluorescence, indicating that LysSXP-2 is non-toxic (Figure 5C). Endolysins typically target the bacterial cell wall, therefore LysSXP01, which lacked a signal peptide and transmembrane domain, cannot traverse the inner cell membrane to exert its lytic effect when expressed alone *in vivo*.

### 3.6 *In vivo* protective efficacy of SXP01 against *Shewanella xiamenensis* infection in crucian carp

**Figure 6:**
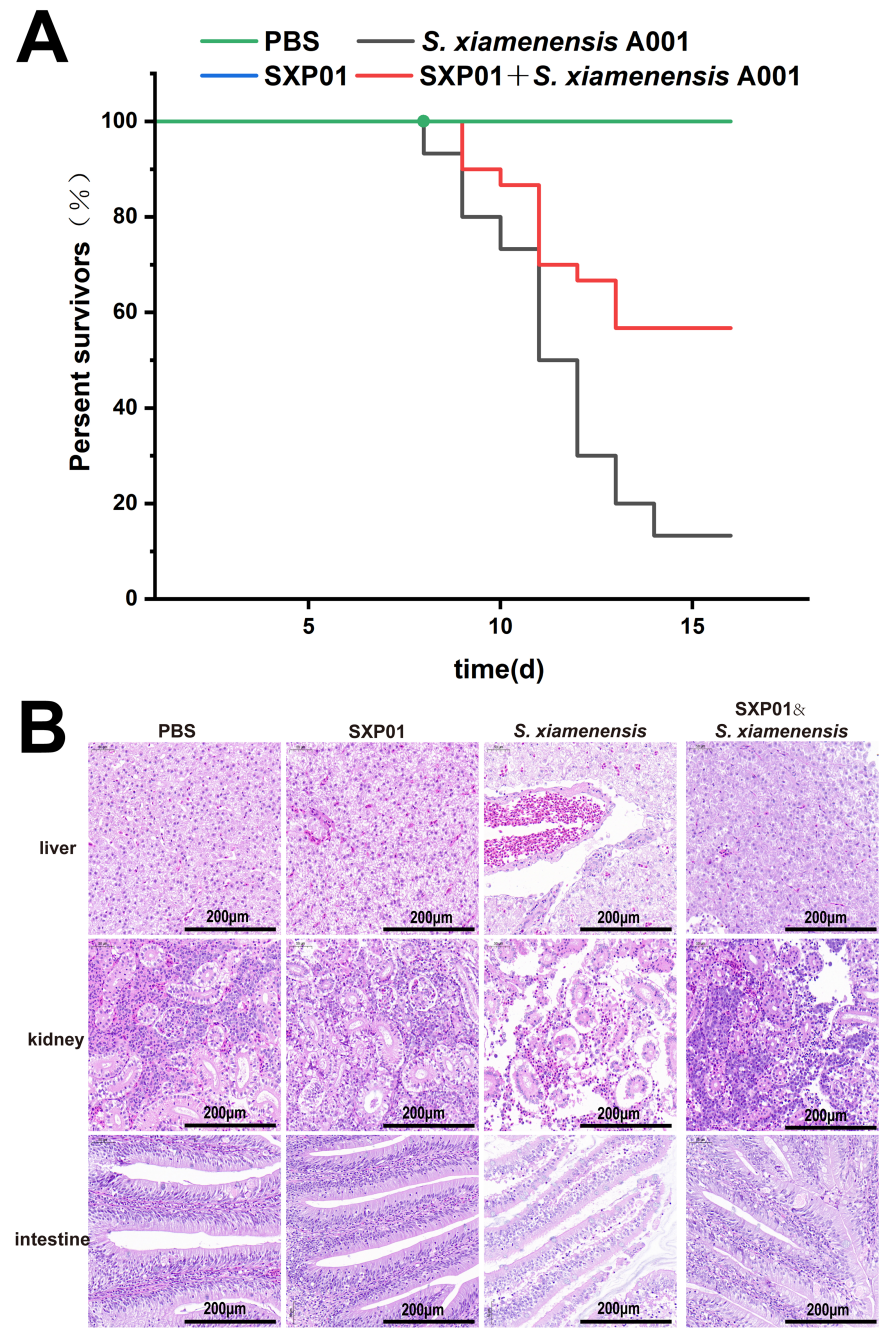
The protective effect of phage SXP01 on crucian carp infected with *S. xiamenensis*. **(A)** The influence of phage on the survival rate of crucian carp infected with *S. xiamenensis*. **(B)** H&E staining observation of the liver, kidney and intestine of crucian carp.

In order to conduct a more in-depth analysis of the effectiveness of SXP01 as an antibacterial agent within the field of aquaculture, we embarked on a study to explore its protective impact on crucian carp that had been infected with *S. xiamenensis*. Our findings revealed that none of the crucian carp in the control group, which had been injected with either phosphate buffer solution (PBS) or SXP01, succumbed to the infection (Figure 6A). However, 7 d subsequent to the administration of *S. xiamenensis* A001 by itself, the carp began to perish, exhibiting minor bleeding at the base of their fins and within their abdominal cavities. This led to a final survival rate of merely 13.3% among this group (Figure 6A). In contrast, when the carp were injected with both *S. xiamenensis* A001 and the phage SXP01, the onset of mortality was delayed until the ninth day, and it stabilized after the thirteenth day (Figure 6A). Ultimately, a commendable 56.7% of the crucian carp survived, which was higher than the survival rate of the group that had only been injected with *S. xiamenensis* A001 (Figure 6A). The survival improvement (13.3 to 56.7%) is promising but still moderate which may be due to the timing and dosage of the medication. Histopathological examinations further substantiated these findings, showing that the intestines, kidneys, and livers of crucian carp that had been injected with either PBS or SXP01 displayed no pathological symptoms, with cells being closely and neatly arranged. Conversely, following the injection of *S. xiamenensis* A001 alone, all tissues exhibited significant lesions. In the liver tissue, extensive hepatocellular necrosis was observed, characterized by numerous scattered white foci and a concurrent reduction in viable hepatocytes (Figure 6B). The intestinal epithelial cells appeared disordered and sparsely arranged, with notable detachment of intestinal villi (Figure 6B). Additionally, the lumen structure of the kidney tissue was severely compromised, and renal cell density was significantly reduced (Figure 6B). Additionally, compared to bacterial infection alone, the application of SXP01 phage post-bacterial infection resulted in a significant reduction in necrotic areas and structural tissue damage, particularly in the liver and intestines. Although the cell density in the kidney tissue was lower and the tissue structure was more loosely arranged compared to PBS group, the improvement was still remarkably significant when compared to the condition after bacterial infection alone (Figure 6B).

## 4 Discussion

Bacteriohages, as antibacterial agents, have attracted considerable interest due to their unique capability to selectively and swiftly eliminate host bacteria, all while sparing the environment and non-target organisms from any harm (Mondal et al., 2022). A multitude of studies have shown that phages can emerge as a viable alternative to traditional antibiotics in the battle against bacterial infections (Almeida et al., 2016; Janelidze et al., 2022; Li et al., 2022). The isolation and characterization of bacteriophage SXP01 underscore its potential as a highly specific biocontrol agent against *S. xiamenensis* A001, a pathogen of significance in aquaculture. The narrow host spectrum of SXP01, restricted exclusively to its host bacterium, aligns with the growing emphasis on precision in phage therapy to minimize collateral damage to non-target microbiota. This specificity is particularly advantageous in aquaculture ecosystems, where preserving beneficial bacterial communities is critical for maintaining water quality and host health.

Morphologically, classification of SXP01 within the Myoviridae family, characterized by its icosahedral head and contractile tail, correlates with its observed lytic efficiency, as Myoviridae phages are often associated with robust host lysis due to their tail structure facilitating DNA injection (Mardiana et al., 2022). The one-step growth curve parameters of SXP01 reflect a replication cycle comparable to other Myoviridae phages, while the optimal MOI of 0.1 suggests efficient infection dynamics at low phage-to-host ratios, a trait favorable for therapeutic dosing strategies. The remarkable stability of SXP01 across a wide range of temperatures (4 ~ 60 °C) and pH values (3~11) highlights its resilience under extreme environmental conditions. This characteristic positions SXP01 as a promising candidate for applications in diverse settings, including industrial processes and aquatic environments with fluctuating physicochemical parameters. The retention of activity following chloroform exposure further underscores its structural integrity, likely due to the absence of a lipid envelope, a characteristic consistent with its classification within the Myoviridae family (Hernández, 2017).

The phylogenetic clustering of SXP01 with Klebsiella phage N1M2 (83.68% genome identity) suggests evolutionary conservation of core structural proteins, such as the major capsid protein, while its genomic novelty underscores the diversity of phages targeting *Shewanella* species. Unlike many phages that contain endolysin-holin two-component lysis systems (Wu et al., 2021), the genome of phage SXP01 reveals the presence of two lysis genes, lysSXP-1 and lysSXP-2, without the corresponding gene encoding holin. Heterologous expression of LysSXP-1 and LysSXP-2 in *E. coli* revealed striking differences in their cytotoxicity. Structural analyses of these proteins revealed that LysSXP-1 contains a transmembrane domain (TMD) and a signal peptide (SP) at the N-terminus, while LysSXP-2 lacks both a signal peptide and a TMD. The presence of the SP indicates that LysSXP-1 may be secreted into the periplasm via the general secretion (Sec) pathway to target the cell wall for lytic activity, akin to LysTP712 encoded by the TP712 genome (Escobedo et al., 2019) and the *Firehammervirus* phage F379 (Zampara et al., 2020). The lack of lytic activity in LysSXP-2 suggests that the genome of bacteriophage SXP01 may harbor unpredicted holin-like genes, potentially linked to the diversity and small molecular weight of holins, as the reported number of holins is less than that of phages (Wang et al., 2000). These findings not only elucidate the functional divergence of phage lysins but also highlight challenges in recombinant production of membrane-targeting enzymes, which may require optimized expression systems to mitigate host toxicity. The arms race between phages and bacteria has led to the emergence of phage resistance, which could compromise the efficacy of phage therapy (Loessner et al., 2020; Hesse et al., 2020; Hampton et al., 2020). In contrast, endolysins do not induce resistance as compared to bacteriophages (Lu et al., 2021), and the modular structure, along with the potent lytic activity of LysSXP-1, positions it as a promising antibacterial agent.

The study of phage therapy in animal models is an essential part from *in vitro* research to clinical treatment. It was reported that *Shewanella* Phage can significantly inhibit bacterial growth in shrimp (Liu et al., 2025). Our *in vivo* trials demonstrated that SXP01 treatment could protect crucian carp against *S. xiamenensis* infection, with a survival rate of 56.7% in phage-treated groups compared to 13.3% in pathogen-only controls. The delayed mortality onset (9 ~ 13 days post-infection) suggests that SXP01 effectively modulates pathogen proliferation, providing a therapeutic window for host recovery. These results align with emerging applications of phage therapy in aquaculture, where targeted pathogen control is prioritized over broad-spectrum antibiotics. However, the incomplete survival rate (56.7%) underscores the need for optimized dosing regimens or combinatorial approaches (e.g., phage-antibiotic synergy) to enhance efficacy.

While SXP01 exhibits significant potential, further studies are necessary to evaluate its long-term stability in aquatic environments, the host immune responses to phage administration, and the potential resistance mechanisms in *S. xiamenensis.* In addressing phage resistance, cocktails may be a promising solution. Research has indicated that, compared to a single phage, the use of phage cocktails can significantly reduce the frequency of bacteria displaying resistance to phage treatment (Yu et al., 2022). Moreover, the ecological safety concerns potentially triggered by the application of bacteriophages should not be overlooked. Genome sequencing and characteristic analysis are critical steps in ensuring their environmental safety. The exclusion of toxin genes or lysogenic control genes can provide significant assurance for the safety of bacteriophages (Cai et al., 2023). Additionally, the structural characterization of LysSXP-1 and LysSXP-2 could provide insights into the design of engineered lysins with enhanced activity or broader specificity. Conducting field trials in aquaculture systems will be critical for assessing scalability and ecological impact, ensuring that the narrow host range of SXP01 translates to sustainable and environmentally safe pathogen control.

In conclusion, this study presents SXP01 as a versatile tool for combating *S. xiamenensis* infections. It integrates genomic novelty, enzymatic synergy, and environmental resilience, thereby establishing a paradigm for phage-based interventions in aquaculture and beyond.

## Data Availability

The genome sequence data of phage SXP01 have been submitted to the GenBank databases under accession number PV593634. The addresses are as follows: https://www.ncbi.nlm.nih.gov/nuccore/PV593634.
