# Supplementary material for: SXP01: a novel bacteriophage for combating Shewanella xiamenensis in aquaculture
Source: Front Microbiol. 2025 Oct 16;16:1652450. doi: 10.3389/fmicb.2025.1652450 (PMC12571629; doi:10.3389/fmicb.2025.1652450)
Supplement: Supplementary file 1 [file Supplementary_file_1.docx]

# Supplementary Figures and Tables

**Table S1.** Primers used in this study.

| **Primers** | **Primer sequences (5'-3')** |
| --- | --- |
| LysSXP-1-F | CCAAGCTTATGAACAGTAAGACCCAAAAC |
| LysSXP-1-R | GCTCTAGATTACAATCCATAACCAACGTA |
| LysSXP-2-F | CGAGCTCATGTCCTTACAGGAAACG |
| LysSXP-2-R | CCCTCGAGTTAAACTAAGCCAAACGCT |

**
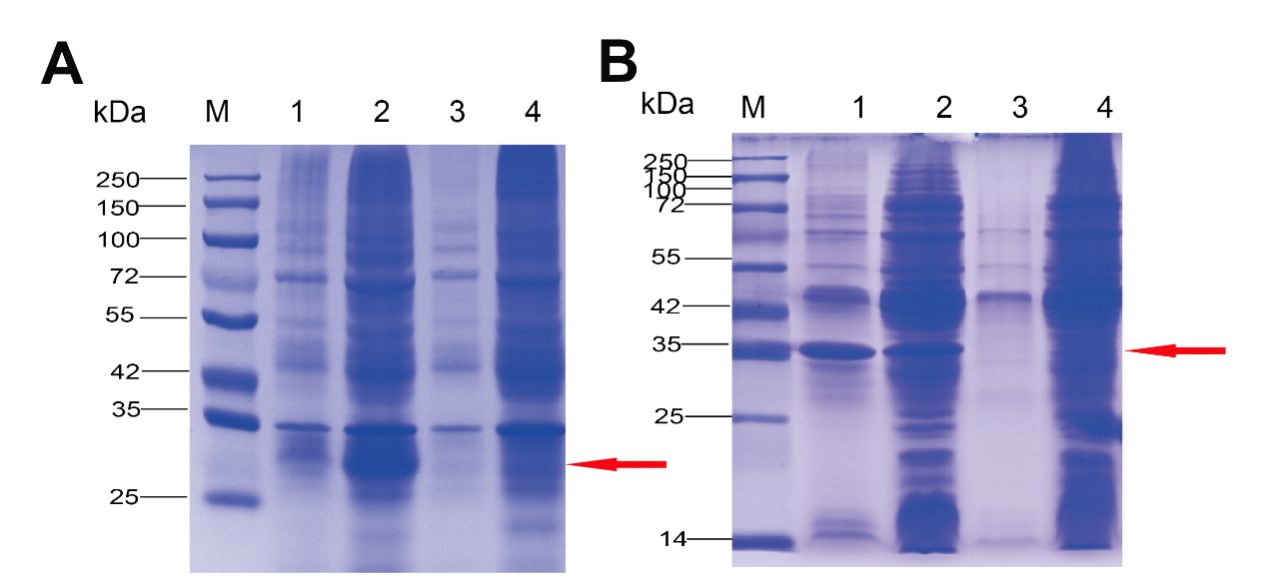
**

**Figure S1.** (A), SDS-PAGE analysis of pET28a-LysSXP-1 after 4 hours of induction. m, markers. Row 1, supernatant of IPTG-induced *E. coli* BL21 (pET28a-LysSXP-1); Row 2, precipitate of IPTG-induced *E. coli* BL21 (pET28a-LysSXP-1); Row 3, supernatant of IPTG-induced *E. coli* BL21 (pET28a); Row 4, precipitate of IPTG-induced *E. coli* BL21(pET28a). (B) SDS-PAGE analysis of pET28a-LysSXP-2 after 4 h of induction. m, marker. Row 1, supernatant of IPTG-induced *E. coli* BL21 (pET28a-LysSXP-2); Row 2, precipitate of IPTG-induced *E. coli* BL21 (pET28a-LysSXP-2); Row 3, supernatant of IPTG-induced *E. coli* BL21 (pET28a); Row 4, precipitate of IPTG-induced *E. coli* BL21 (pET28a). The red arrow represents the expected position of the protein.
